# Draft genome sequence data of methicillin-resistant *Staphylococcus aureus*, strain 4233

**DOI:** 10.1016/j.dib.2024.110492

**Published:** 2024-05-11

**Authors:** Madina Alexyuk, Andrey Bogoyavlenskiy, Yergali Moldakhanov, Kuralay Akanova, Adolat Manakbayeva, Pavel Alexyuk

**Affiliations:** Research and Production Center for Microbiology and Virology, Bogenbay batyr. Str., 105, Almaty 050010, Kazakhstan

**Keywords:** Antibiotic resistance, Nosocomial infection, Illumina sequencing, Genome annotation

## Abstract

*Staphylococcus aureus* is a conditionally pathogenic microorganism and one of the main causative agents of antibiotic resistant nosocomial infections. In immunocompromised people, *S. aureus* infection can cause folliculitis, furuncles, impetigo, osteomyelitis, septic arthritis, sepsis, endocarditis, pneumonia and meningitis. In the presented work, sequencing of a methicillin-resistant *S. aureus*, strain 4233, was performed on the Illumina MiSeq platform, followed by bioinformatics processing and gene annotation using SPAdes, RAST and CARD programs and databases. The submitted genome is a total of 2,790,390 bp long and contains 2759 genes, including 82 RNA genes. 33 % of the genes are functionally significant and represent 25 functional groups. Fourteen genes encoding resistance factors to 14 different types of antibacterial drugs were predicted. The information provided on the genome of *S. aureus*, strain 4233 will be of value in investigating the evolution and formation of antibiotic-resistant forms of *S. aureus*.

Specifications Table

**Table:** 

Subject	Microbiology: Bacteriology
Specific subject area	Microbial genomics
Type of data	Raw and processed sequencing data, genome annotation and phylogenetic analysis. Table, Graph, Figure.
Data collection	Collection: The sample was obtained from hospital wastewater. The sample was cultured on selective media. Gram staining and enzymatic activity were used for identification of isolate. DNA extraction was performed using the PureLink Genomic DNA Mini Kit (Invitrogen, USA). The Miseq Illumina sequencing platform was used to generate paired-end reads. Software: The entire genome sequence was assembled using SPAdes 3.12.0 assembler in Geneious Prime. Genome sequences were annotated using the GenBank PGAP annotator robot and deposited at NCBI. The Resistance Gene Identifier was used for screening of antimicrobial resistance genes. MAFFT in Geneious Prime was used for phylogenetic analysis.
Data source location	Research and Production Center for Microbiology and Virology, Almaty, Kazakhstan. • City/Region: Almaty • Country: Kazakhstan • Latitude and longitude for collected samples: 43°15′14.2″N 76°57′11.1″E
Data accessibility	Repository name: GenBank: Data identification numbers: BioProject Accession Number: PRJNA1014944, NCBI SRA Accession Number: SRS20908588, NCBI GenBank Accession Number: NZ_CP134071.1 The direct URL to the data: https://www.ncbi.nlm.nih.gov/bioproject/1014944 https://www.ncbi.nlm.nih.gov/sra/?term=SRS20908588 https://www.ncbi.nlm.nih.gov/nuccore/NZ_CP134071.1

## 1 Value of the Data

• The presented data provide information on the whole-genome sequence of methicillin-resistant *S. aureus* isolate, strain 4233 isolated from the wastewater of the hospital of Almaty city, Kazakhstan.
• Methicillin-resistant pathogenic strains of *S. aureus* can cause dangerous diseases that are difficult to treat. In this regard, the presented data may contribute to a better understanding of genome variability, adaptive capacity, resistance mechanisms, and genetic differences between *S. aureus* strains and contribute to the development of more effective control and prevention strategies against this pathogen.
• The presented data can be useful for the scientific and medical community, find their application in research on microbiology, medicine, molecular biology, bacterial genomics.
• The obtained data are publicly available in NCBI databases and can be used as a basis for evolutionary research using reverse genetics methods, for studying the pathways of resistance formation in pathogenic forms of *S. aureus* and identification of genetic determinants of pathogenicity.

## 2 Background

*S. aureus* is a conditionally pathogenic microorganism and one of the main causative agents of nosocomial infections. In immunocompromised people, *S. aureus* can cause the development of both mild inflammatory processes and serious diseases [1,2]. In addition, *S. aureus* can lead to the development of food poisoning if enter the body with food [3,4].

The fight against *S. aureus* infections is complicated by the development of multiple drug resistance, which significantly reduces the effectiveness of antibiotic therapy. According to WHO data, from 20 % to 80 % of hospital-acquired infections are caused by antibiotic-resistant strains of *S. aureus* [5].

In order to solve the problem of antibiotic resistance, it is necessary to make a comprehensive study of drug-resistant bacterial strains. It is known that methicillin-resistant *S. aureus* strains responsible for the development of hospital-acquired infections are capable of genetic adaptation [6,7]. Therefore, studying the genome of strains of this microorganism isolated in different countries and continents will contribute to the understanding of the complex host-pathogen interactions and pave the way for the treatment of people infected with MRSA today. In our study, we present a draft genome of methicillin-resistant *S. aureus* isolated near a hospital in Kazakhstan.

## 3 Data Description

**Fig. 1:**
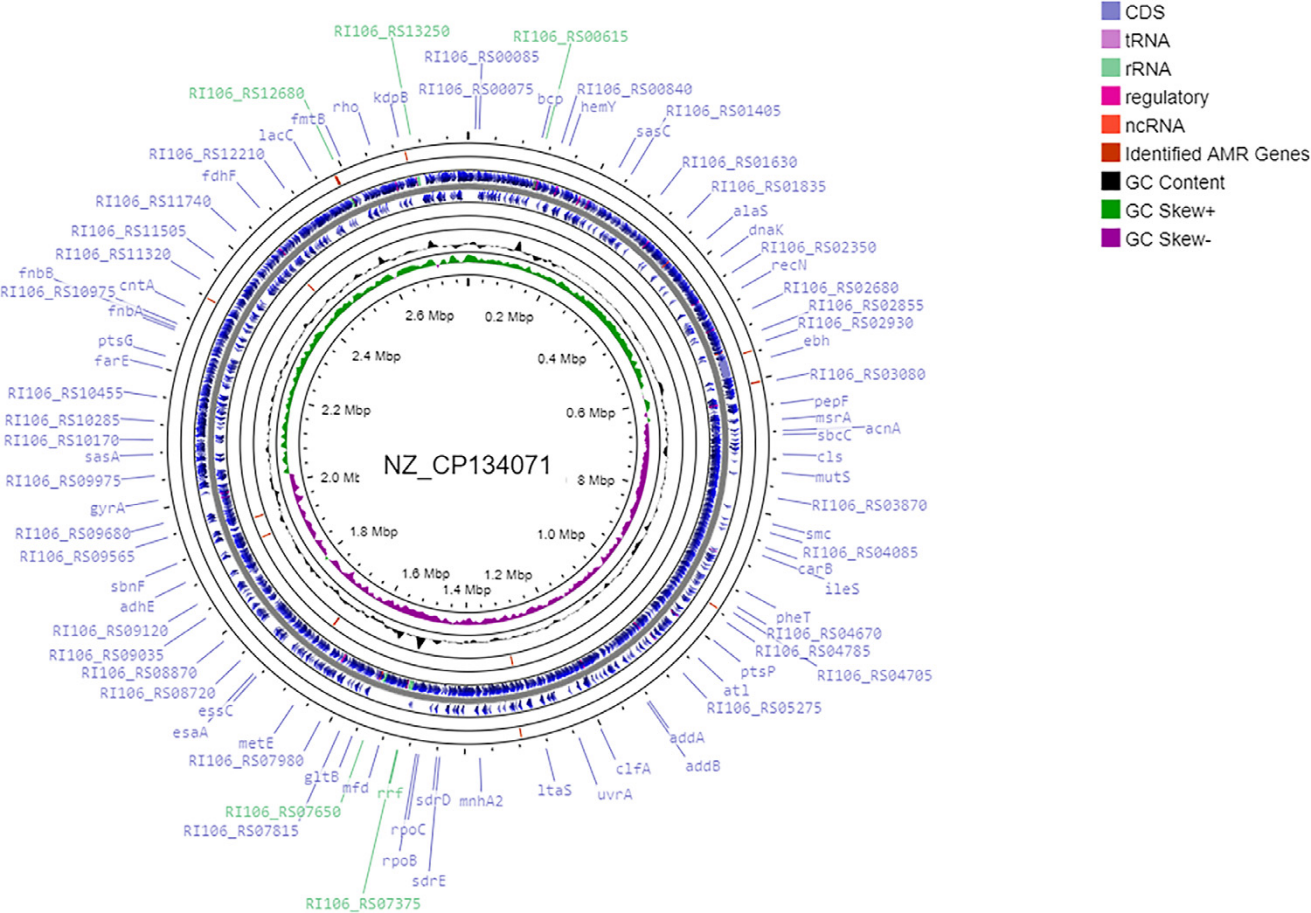
The genome map of *S. aureus* strain 4233 built using the CGView server (https://proksee.ca/ assessed on 11th March 2024). The blue arrows represent CDSs; green peaks represent GC-skew+; purple represents GC-skew-; and black peaks represent G+C content.

This work presents a draft genome sequence of a methicillin-resistant *S. aureus*, strain 4233 (Table 1, Fig. 1). This strain was isolated from the wastewater of the Central Clinical Hospital JSC in Almaty. The presented genome is a total of 2790,390bp long and contains 2759 genes, including 82 RNA genes.

**Table 1:** Genome characteristics of *S. aureus*, strain 4233.

Genome size (bp)	2790,390
GC% content	32,8 %
Number of contigs ≥ 1000 bp	276
Max Lengh of contig	116,069
N50 length (bp)	29,734
Genes (total)	2759
CDSs (total)	2724
Genes (RNA)	82
tRNAs	59

**Fig. 2:**
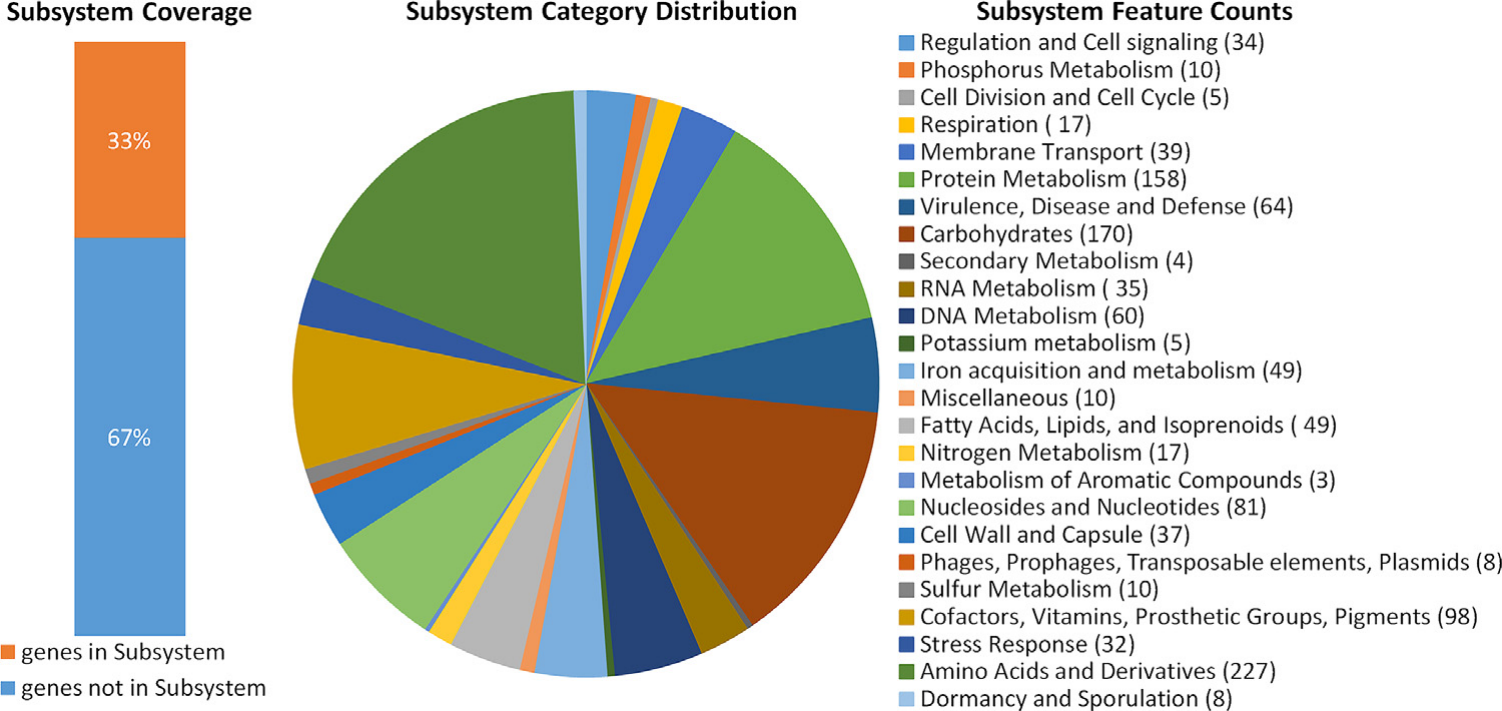
Subsystem statistics information on genome *S. aureus*, Strain 4233 obtained using RAST annotation. The subsystems categories and corresponding counts are presented in the legend.

Annotation in the RAST program enabled us to determine that 33 % of the genes were functionally significant and represented 25 functional groups (Fig. 2) [8]. The largest number of genes, more than 100, belonged to the following groups: Amino Acids and Derivatives, Carbohydrates and Protein Metabolism - 227, 170 and 158 genes, respectively. The least number of genes, less than 10, belonged to the groups Transposable Elements (Phages, Prophages, Plasmids), Dormancy and Sporulation - 8 genes, Cell Di-vision and Cell Cycle and Potassium metabolism - 5 genes, Secondary Metabolism and Metabolism of Aromatic Compounds - 4 and 3 genes, respectively. In the remaining 16 functional groups, the number of genes varied from 10 to 98.

**Fig. 3:**
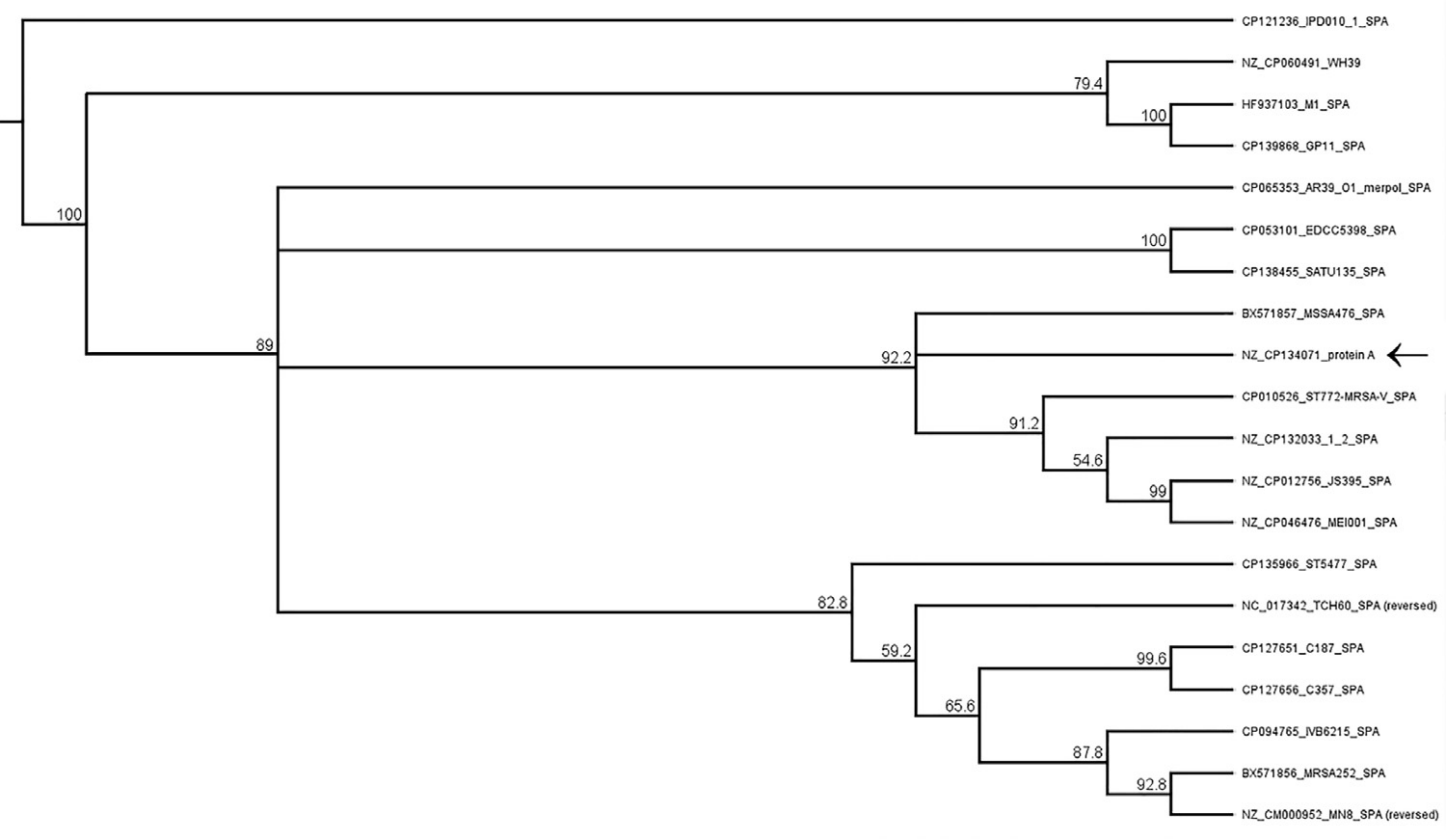
Phylogeny of dominant *S. aureus* strains among nosocomial infections based on the protein A sequence model.

To perform phylogenetic analysis, we used the sequence of protein A (Fig. 3), which is one of the main genetic markers of *S. aureus* [9]. The selection of strains for comparison was chosen to include the strains of the pathogen most frequently encountered in clinical samples and samples isolated from animals [10,11].

It is shown that three branches of evolution are clearly distinguished among the dominant groups of clinical strains of S. aureus belonging to human and animal representatives. The studied strain clearly corresponds to the dominant group of human strains belonging to groups ST 45, 30 and 15.

The presence of antibiotic resistance genes in the genome of *S. aureus*, strain 4233 was determined using the Comprehensive Antibiotic Resistance Database (CARD; card.mcmaster.ca). The predicted resistance genes in the genome are presented in Table 2. Fourteen genes encoding resistance factors to 14 different types of antibacterial drugs were found.

**Table 2:** Predicted antimicrobial resistance genes of *Staphylococcus aureus*, Strain 4233.

RGI Criteria	ARO Term	Detection Criteria	AMR Gene Family	Drug Class	Resistance Mechanism	% Identity of Matching Region	% Length of Reference Sequence
Perfect	arlR	protein homolog model	major facilitator superfamily (MFS) antibiotic efflux pump	fluoroquinolone antibiotic, disinfecting agents and antiseptics	antibiotic efflux	100.0	100.00
Perfect	arlS	protein homolog model	major facilitator superfamily (MFS) antibiotic efflux pump	fluoroquinolone antibiotic, disinfecting agents and antiseptics	antibiotic efflux	100.0	100.00
Perfect	*S. aureus* norA	protein homolog model	major facilitator superfamily (MFS) antibiotic efflux pump	fluoroquinolone antibiotic	antibiotic efflux	100.0	100.00
Perfect	mgrA	protein homolog model	ATP-binding cassette (ABC) antibiotic efflux pump, major facilitator superfamily (MFS) antibiotic efflux pump	fluoroquinolone antibiotic, cephalosporin, penam, tetracycline antibiotic, peptide antibiotic, disinfecting agents and antiseptics	antibiotic efflux	100.0	100.00
Perfect	mepR	protein homolog model	multidrug and toxic compound extrusion (MATE) transporter	glycylcycline, tetracycline antibiotic	antibiotic efflux	100.0	100.00
Strict	norC	protein homolog model	major facilitator superfamily (MFS) antibiotic efflux pump	fluoroquinolone antibiotic, disinfecting agents and antiseptics	antibiotic efflux	70.86	100.22
Strict	PC1 beta-lactamase (blaZ)	protein homolog model	BlaZ beta-lactamase	penam	antibiotic inactivation	95.02	100.00
Strict	norC	protein homolog model	major facilitator superfamily (MFS) antibiotic efflux pump	fluoroquinolone antibiotic, disinfecting agents and antiseptics	antibiotic efflux	99.13	100.00
Strict	*S. aureus* FosB	protein homolog model	fosfomycin thiol transferase	phosphonic acid antibiotic	antibiotic inactivation	98.56	100.00
Strict	sdrM	protein homolog model	major facilitator superfamily (MFS) antibiotic efflux pump	fluoroquinolone antibiotic, disinfecting agents and antiseptics	antibiotic efflux	99.78	100.00
Strict	sepA	protein homolog model	small multidrug resistance (SMR) antibiotic efflux pump	disinfecting agents and antiseptics	antibiotic efflux	96.18	100.00
Strict	*Staphylococcus aureus* LmrS	protein homolog model	major facilitator superfamily (MFS) antibiotic efflux pump	macrolide antibiotic, aminoglycoside antibiotic, oxazolidinone antibiotic, diaminopyrimidine antibiotic, phenicol antibiotic	antibiotic efflux	99.38	100.00
Strict	kdpD	protein homolog model	kdpDE	aminoglycoside antibiotic	antibiotic efflux	98.87	100.00
Strict	vanT gene in vanG cluster	protein homolog model	glycopeptide resistance gene cluster, vanT	glycopeptide antibiotic	antibiotic target alteration	33.88	53.65

Methicillin-resistant strains of *S. aureus* are widespread throughout the world and account for up to 1 % of all isolated strains of the microorganism [12]. Their ability to form epidemically significant variants due to genome adaptability creates serious challenges for public health. Therefore, the determination of genome features of newly isolated strains is important for studying the evolution of this group of microorganisms. In our research we presented a draft genome of *S. aureus*, strain 4233, isolated from the sewage of the Central Clinical Hospital in Almaty, Kazakhstan. The peculiarity of the studied strain is that 14 additional resistance genes to antibiotics with different action mechanisms were found in its genome.

Thus, the draft genome of *S. aureus*, Strain 4233 can serve as an aid for researchers studying the spread of MRSA in different countries.

## 4 Experimental Design, Materials and Methods

### 4.1 Sample collection and isolation of *staphylococcus*

The water sample was obtained from hospital wastewater in Almaty, Kazakhstan (43.24776402 N 76.94454847 E). The wastewater sample was placed in a sterile 500 ml bottle and transported in a refrigerated hold at +4 °C to the laboratory for analysis. Petri dishes containing mannitol-salt agar (CondaLab, Spain) were inoculated with 1 ml of the wastewater sample and incubated for 18–24 h. At the end of the incubation time, a single colony with typical *S. aureus* morphology was selected for further study. Further identification of *S. aureus* was based mainly on the colony morphology, Gram staining and determination of catalase and coagulase activity. To detect MRSA, the bacterial suspension of *S. aureus* was plated on chromogenic MRSA agar with cefoxitin MRSA supplement (CondaLab, Spain) and incubated aerobically at 35±2 °C for 24–48 h. Methicillin resistance was determined by the growth of a bacterial culture in the form of blue-colored colonies [13,14].

The purity of the isolated strains was confirmed using the standard microbiological method. The culture was stored in 20 % glycerol stock at −80 °C.

#### 4.1.1 DNA isolation, genome sequencing, assembly, and annotation

For genomic DNA isolation, *S. aureus*, strain 4233 was grown in 5 ml TSB (Condalab, Spain) at 37 °C for 18–20 h. After that bacterial cells were precipitated by centrifugation at 6000 rpm for 30 min. The supernatant was discarded and the cells were resuspended in sterile phosphate buffered saline and subjected to genomic DNA isolation using the PureLink™ Genomic DNA Mini Kit (ThermoFisher Scientific, Waltham, MA, USA) according to the supplierʼs instructions. DNA quantification was performed using a Qubit® 3.0 fluorometer (Invitrogen, USA) and the Qubit dsDNA High Sensitivi-ty Kit (Invitrogen, USA).

*The genome sequencing of S. aureus*, strain 4233 was performed using Illumina Miseq platform and Miseq kit v3 (Illumina, Cambridge, UK) which allows to obtain 300 bp long paired-end reads. The library was obtained using the Nextera XT DNA library preparation kit (Illumina, Cambridge, UK).

Raw read adapters were trimmed using Trimmomatic 0.38.0 software [15]. Sequences of low quality (<Q30) were removed, after which, the remaining reads were on average 50–250 bp in length. De novo assembly of quality-controlled reads was performed using SPAdes 3.12.0 [16]. The quality of the assembled genome was determined by comparison with the reference genome using the Geneious Prime program version 2023. Annotation of the assembled genome was carried out using the NCBI Prokaryotic Genome Annotation Pipeline (PGAP), GeneMarkS-2+, RAST and Bacta [17,18]. The Resistance Gene Identifier (RGI v5.2.0) was used to detect AMR genes [19]. For phylogenetic analysis, we used the pro-gram for constructing trees based on the nearest neighbor after performing the MAFFT (Multiple Alignment using Fast Fourier Transform) alignment, which is part of the Geneious Prime 2023 software.

The raw genome sequencing data of Illumina MiSeq were submitted to NCBI SRA database in FASTQ format: SRS20908588, with BioSample: SAMN37344193, under BioProject PRJNA1014944. The assembled genome is available in the NCBI GeneBank under NZ_CP134071.1 [20].

## Limitations

Not applicable.

## Ethics Statement

Work did not include animal experiments or data collected from social media platforms or human subjects.

## Funding

This work was supported by 10.13039/501100004569Ministry of Science and Higher Education of the Republic of Kazakhstan, grant number AP14870277.

## CRediT authorship contribution statement

**Madina Alexyuk:** Investigation, Methodology, Data curation, Writing – review & editing. **Andrey Bogoyavlenskiy:** Methodology, Software, Writing – original draft. **Yergali Moldakhanov:** Investigation. **Kuralay Akanova:** Investigation. **Adolat Manakbayeva:** Investigation. **Pavel Alexyuk:** Conceptualization, Software, Validation, Writing – review & editing.

## Data Availability

Genome sequencing (Original data) (GenBank)
